# Meta-Wearable Antennas—A Review of Metamaterial Based Antennas in Wireless Body Area Networks

**DOI:** 10.3390/ma14010149

**Published:** 2020-12-31

**Authors:** Kai Zhang, Ping Jack Soh, Sen Yan

**Affiliations:** 1 School of Information and Communications Engineering, Xi’an Jiaotong University, Xi’an 710049, China; kaizhang2018@stu.xjtu.edu.cn; 2 Advanced Communication Engineering (ACE) Centre of Excellence, Universiti Malaysia Perlis, Kangar 01000, Malaysia; pjsoh@unimap.edu.my; 3 Faculty of Electronic Engineering Technology, Universiti Malaysia Perlis, Arau 02600, Malaysia

**Keywords:** metamaterial, wearable antenna, metasurface, wireless body area network

## Abstract

Wireless Body Area Network (WBAN) has attracted more and more attention in many sectors of society. As a critical component in these systems, wearable antennas suffer from several serious challenges, e.g., electromagnetic coupling between the human body and the antennas, different physical deformations, and widely varying operating environments, and thus, advanced design methods and techniques are urgently needed to alleviate these limitations. Recent developments have focused on the application of metamaterials in wearable antennas, which is a prospective area and has unique advantages. This article will review the key progress in metamaterial-based antennas for WBAN applications, including wearable antennas involved with composite right/left-handed transmission lines (CRLH TLs), wearable antennas based on metasurfaces, and reconfigurable wearable antennas based on tunable metamaterials. These structures have resulted in improved performance of wearable antennas with minimal effects on the human body, which consequently will result in more reliable wearable communication. In addition, various design methodologies of meta-wearable antennas are summarized, and the applications of wearable antennas by these methods are discussed.

## 1. Introduction

**Figure 1:**
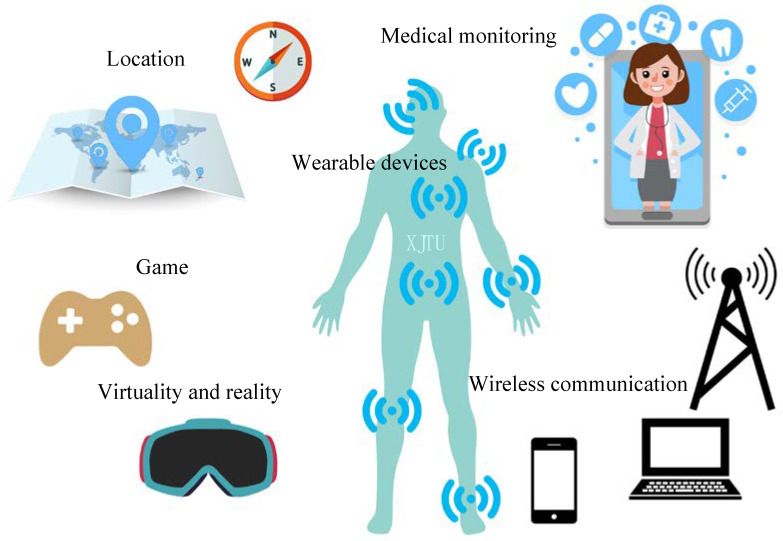
A Wireless Body Area Network (WBAN) and its applications [1,8,9,10,11,12,13,14,15,16].

The emergence of the Internet has brought about tremendous changes in the communication of human. Wireless Body Area Network (WBAN) is one of the emerging technologies that is capable of enabling the communication between people and things. The development of body area network will result in a more context-aware and personalized communication in an intelligent wireless environment. WBAN is a network distributed around human, which is mainly used to detect and transmit physiological data of users, and cooperate with other networks to integrate the human into the overall network [1,2,3,4,5]. Through WBAN, communication and data synchronization can take place to complete the other communication networks, such as wireless sensor networks and mobile communication networks [1,2,6,7]. An important application of WBAN is in healthcare, where the body area network can transmit physiological information obtained from patients through various physiological sensors, such as blood pressure, blood sugar concentration, temperature, weight, and heartbeat [1,8,9,10] to the hospital’s medical monitoring equipment or the user’s personal mobile terminal [11]. In entertainment, a personal media device with high speed communication capability will enable augmented/virtual/mixed reality interaction with users, and wirelessly communicate with a device such as glasses [12] or headset [13]. In military applications, WBAN can provide personal location and mobile communications by a helmet [14,15] or a smart watch [16], etc. Figure 1 shows some typical applications of WBANs.

Wearable antennas, as a vital component in WBAN systems, enable wireless communication with other devices on or off human bodies [17,18,19,20,21,22,23,24,25,26,27,28,29,30,31,32,33,34,35,36,37,38,39,40,41,42,43]. Compared to traditional antennas, the design of wearable antennas are facing many development bottlenecks: The electromagnetic coupling between the human body and the antenna, the varying physical deformations, the widely varying operating environments, and limitations of the fabrication process [27,28,29,30,31,32,33]. Further, the requirements for these wearable antennas include mechanical robustness, low-profile, lightweight, user comfort, fabrication simplicity [17,18,19,20,21,22,23,24], wideband [25,26], and multiband [20,27,29]. Thus, advanced design methods and techniques are urgently needed to address these problems and demands of wearable antennas. In recent years, there has been much literature reporting the fabric material manufacturing and treatment: Embroidered fabric material, sewn textile materials, woven fabrics, materials that are not woven, knitted fabrics, spun fabrics, braiding, coated fabrics through/lamination, printed fabrics, and chemically treated fabrics [18,19]. Furthermore, novel forms of flexible devices such as a fully inkjet-printed antenna [30,31], a polydimethylsiloxane (PDMS)-based antenna [21,22], embroidery [32], and a silicone-based antenna [33], and devices combined with new design methods such as substrate-integrated waveguide (SIW) technology [34], miniature feeding network [23], magneto-electric dipole [35], characteristic mode theory [27,36,37], textile-type indium gallium zinc oxide (IGZO)-based transistors [30], and thin-film transistor technologies [24], are presented for special application scenarios. Furthermore, miniaturization methods, such as inductor/capacitor-loaded antennas [38,39,40], loop antennas [41], and planar inverted F antennas (PIFA) [42,43] are involved in WBAN devices design, which is helpful to improve the design flexibility of the wearable antennas.

Metamaterials are widely defined as an artificial periodic structure, in which the length of the unit cell *p* is much smaller than the guided wavelength *λ_g_*, with unusual properties not available in nature in the electromagnetic field [44,45,46]. The use of metamaterials has been hugely successful in adapting conventional antenna designs into a wearable form, including composite right/left-handed transmission lines (CRLH TLs) based antennas [45,46,47,48,49,50,51,52,53,54], zero-order antennas [55,56], metamaterials-inspired antennas [32,57], artificial magnetic conductor (AMC) [58,59,60,61], electromagnetic band-Gap (EBG) [62,63,64,65], and High-Impedance Surface (HIS) [16]. Wearable antennas have been designed with properties such as multiple band operations [48,55], multiple functionalities [66,67,68,69], and gain enhancement [70,71] while maintaining a low profile and compact size [56,72,73] due to the development of the electromagnetic metamaterials tech. Besides the methods above, characteristic mode theory is also an excellent analysis approach to study the metamaterials-based antennas [74,75,76], and it improves the efficiency of antennas design in WBAN. Several initial attempts have shown that it is an effective method to design wearable antennas based on metamaterials.

This manuscript reviews the recent progress in the field of wearable antennas with metamaterials. Firstly, several designs based on CRLH TLs, a typical 1D metamaterial, will be summarized. The designs easily realize a compact size or dual-band operating bands. Next, the designs based on 2D metamaterials, i.e., metasurfaces, will be discussed. The main advantages of these antennas are the low-profile and the wide operating band. Finally, several antennas with reconfigurability will be shown based on reconfigurable metamaterials. The uniqueness of this review is that the design methodologies of the wearable antennas based metasurface are summarized in recent years, and the merits and drawbacks of the various approaches to design wearable metasurface antennas are compared and discussed. Furthermore, the applications of wearable metasurface antennas designed by these methods are described, satisfying the diversified demands in our life. Besides, the challenges and the directions in metamaterial-based antennas in WBAN in the future are emphasized.

## 2. Wearable Antennas Based on Composite Right/Left-Handed Transmission Lines (CRLH TLs)

### 2.1. Wearable Electrical Small Antennas (ESAs) Based on Zeroth-Order Resonance (ZOR)

**Figure 2:**
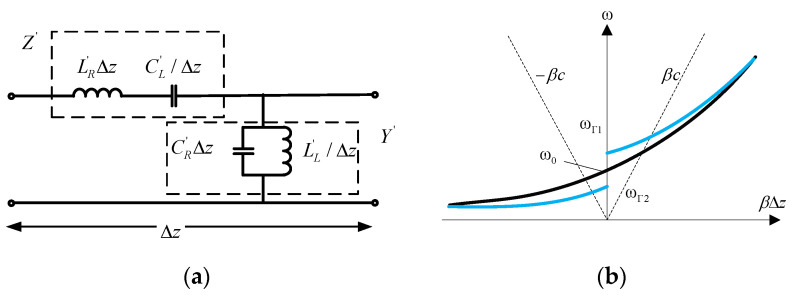
(**a**) The circuit model of a unit cell of with composite right/left-handed transmission lines (CRLH TLs) consists of two structures: A parallel resonant topology and a series resonant topology, and (**b**) the dispersion curve is obtained when the electromagnetic wave through the circuit of the unit cell with a length of Δ*z*, where the ω_Γ1_ and ω_Γ2_ are the two frequencies of zero-order resonance.

CRLH TLs is one form of electromagnetic metamaterials, which usually is presented as a uniform periodic structure. A unit cell of a typical CRLH TLs is shown in Figure 2a, including a parallel resonant topology and a series resonant topology. According to this, the dispersion relation is drawn in Figure 2b, where the Δ*z* is the length of the unit cell. The intersections between the dispersion curve and ω-axis mean that the resonator can generate the zero-order resonances.

Generally, there are three kinds of forms in CRLH zero-order resonance antennas: Epsilon-negative resonance (ENR) [56,68,69,72,73], Mu-negative resonance (MNR) [14,48,49,50,51,52], and dual-negative resonance (DNR) [53,54,77,78,79,80]. ENR is enabled by loading shunt inductance in the equivalent circuit of a conventional transmission line, and the effective permittivity of the unit cell is negative. MNR indicates that the series capacitance is loaded in the equivalent circuit of the conventional transmission line, and the effective permeability of the unit cell is negative. DNR represents that both the effective permittivity and effective permeability are negative.

**Figure 3:**
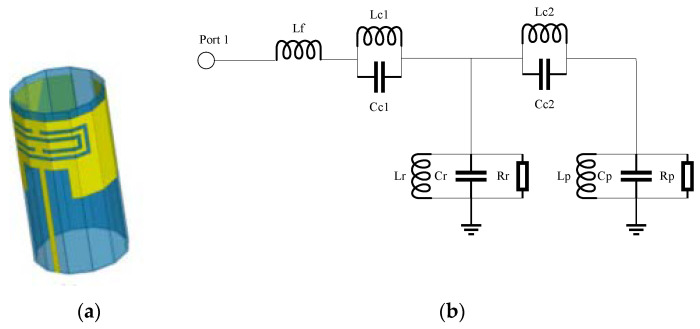
A compact omnidirectional self-packaged patch antenna with complementary split-ring resonator loading for wireless endoscope applications [77]. (**a**) The model of the package state of the antenna. (**b**) Equivalent circuit of the antenna.

Cheng et al. designed a patch loaded with a complementary split-ring resonator (CSRR), which is fabricated on a flexible substrate and folded in a cylindrical shape, forming a self-packaged folded patch antenna as shown in Figure 3a [77]. The advantage of this antenna is that the cavity of the package can shield the electromagnetic wave and alleviate electromagnetic interference (EMI). The loaded CSRR patch is modeled as an RLC tank, and the microstrip components consisting of the patch and the feeding line are represented by the capacitive and the inductive parameters, respectively, which can be modeled as an LC tank, whose equivalent circuit is shown in Figure 3b. Essentially, this antenna employed the principle of DNR. There exists both negative equivalent permittivity and equivalent permeability, and the performances detail are listed in Table 1.

Another DNR flexible antenna without via-holes is designed by Kim et al. [78]. The printed circuit board can be represented as the unit cell of CRLH TLs circuit model in Figure 2. This via free antenna can significantly alleviate the complexity and fabrication cost. The proposed structure is involved with a vertically polarized antenna with a monopole-like radiation pattern, which is suitable for many commercial and personal electronics. An air bridge is introduced for reducing loss, and the measured radiation efficiency of the planar ZOR antenna confirms a maximum improvement of nearly 10% at the central operating frequency of 2.37 GHz.

Wearable antennas do not only require a good radiation performance and a miniature dimension but also need to have a low Specific Absorption Rate (SAR) for human body tissue. In 2010, Jung et al. presented a flexible DNR zeroth-order antenna, which introduced four CRLH TL unit cells and possessed a zero-phase constant, representing a minimization [79]. This antenna is fabricated on Rodgers RO3003 with a thickness of 0.5 mm, whose electoral length of the unit cell is designed to be 0.04 *λ_g_*. Its ZOR frequency is 2.45 GHz and the 10dB bandwidth is 6.5%. However, this antenna is not a good candidate for a wearable device because the most energy radiated from it would be absorbed by users’ bodies. It may lead to a high SAR value when mounted on human bodies. In order to overcome this defect, Lee et al. proposed a wrist antenna as the same principle, which is designed using a Coplanar Waveguide Ground (CPWG) technique in 2011 [80]. The performance of the proposed antenna is insensitive to the on-body condition because the ground plate on the bottom of the substrate can actually play an isolator. The finite size ground plane can provide high isolation between the antenna and human tissue and promote the antenna property of radiation.

In the wearable antenna field, the research on ENR and MNR zero-order mode antenna is rare. However, the proper utilization of them on other kinds of antennas has been published [48,49,50,51,52,56,68,73]. Considering the difficult implementation of both the capacitors and the inductors, new types of metamaterials that support only ENR or MNR mode is a new direction in wearable ZOR antennas. Compared with DNR antennas, ENR antennas can be utilized as a simple structure antenna [56]. For the foreseeable future, both the ENR and the MNR antennas will have a widely use in wearable designs, in addition to DNR structures.

### 2.2. Wearable Dual-Band Patch Antennas Based on CRLH TLs

**Figure 4:**
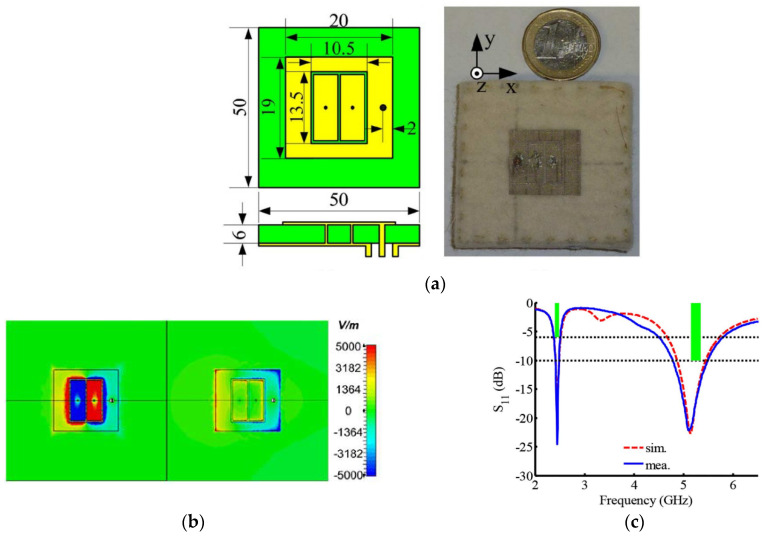
A compact all-textile dual-band antenna loaded with metamaterial-inspired structure [57]. (**a**) The simulation model and the prototype (in mm). (**b**) The E-field distribution at 2.45 GHz (−1 mode) and 5.2 GHz (+1 mode) in z-direction. (**c**) S11 curves of the wearable antenna in simulation and measurement.

**Figure 5:**
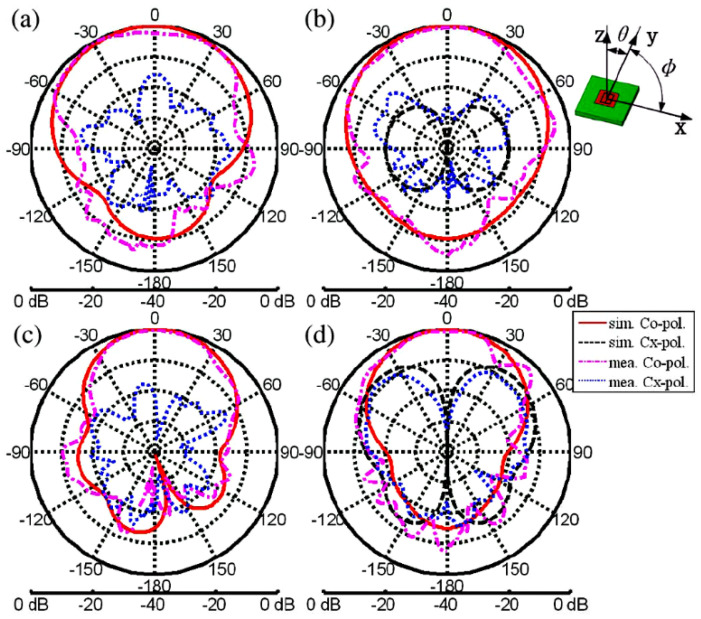
Simulated and measured radiation patterns in the (**a**) lower band in-plane, (**b**) upper band in-plane, (**c**) lower band in-plane, and (**d**) upper band in-plane [57].

Two methods are generally used to design a wearable dual-band patch antenna based on CRLH TL: By either using two different resonant orders or using dual-zero resonances. The method of using two different resonances refers to the two bands either being represented by negative modes, zeroth-order modes, positive modes, or the combinations of them. Yan et al. presented two wearable antennas loaded with CRLH TL topologies using the principle of two symmetric resonant orders [57,58]. More specifically, the two resonances are with the same mode number but with different signs (±n). The substrates of the proposed wearable antennas are a 3 mm and a 6 mm thick felt with relative permittivity of 1.3 and loss tangent of 0.044. The two resonances are observed at 2.45 and 5.15 GHz, corresponding to the first order of the negative mode and the positive mode. This configuration results in a similar field distribution and thus similar radiation patterns in the two bands. The antenna model and its performances in S11 and radiation patterns are shown in Figure 4 and Figure 5.

In 2018, Lee et al. proposed a compact dual-band patch antenna using MNR for a smart helmet operating in the 2.4 GHz and 5 GHz bands [14]. This antenna consists of two MNR loops, which are coupled to each other to excite a two-order resonance using the outer MNR loop. At 2.4 GHz, a ZOR mode is introduced by the current on the outer loops of the two MNRs, which flows in the same direction with no significant phase difference. On the other hand, at 5 GHz, the current on the outer loop flows in the opposite direction, indicating the first mode. The two modes will generate different radiation patterns, which are suitable for on- and off-body communications using a single radiator.

**Figure 6:**
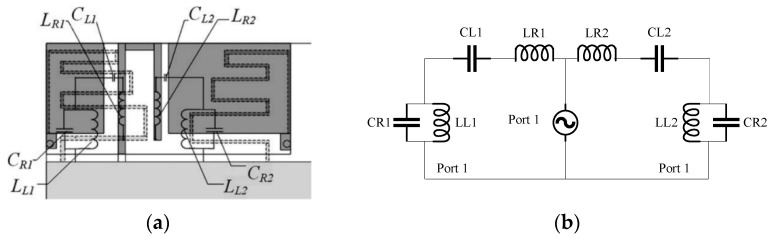
A compact multiband antenna employing dual-band CRLH TL for smart mobile phone application [53]. (**a**) The schematic diagram of the antenna with the circuit parameters. (**b**) The equivalent circuit of this antenna.

There is very limited literature on structures providing dual-zero resonances. Nonetheless, a compact dual-band antenna using dual zeroth-order resonance for smart mobile phone application was proposed in [53]. The two CRLH TL structures, as shown in Figure 6, are excited using a folded feedline, and each contributed a ZOR mode, in which each band being able to be tuned flexibly. Here, the performances of a part of the CLRH TLs based antennas are listed in Table 1 for comparison.

## 3. Wearable Antennas Based on Metasurfaces

The in-phase reflection characteristic of Artificial Magnetic Conductor (AMC) structures can effectively reduce backward radiation of the wearable antenna while maintaining a low antenna profile, improve gain, and reduce the coupling between the antenna and the human body. Thus, recently, metasurface-based wearable antennas have become a popular method to improve the radiation behavior for wearable antennas.

### 3.1. Single-Band Wearable Antennas with Metasurfaces

It is commonly known that the radiated energy from wearable antennas may potentially be harmful to human health [81,82]. Due to its proximity to the human body, the biological tissues in the human body may be affected by the radiated energy on the one hand, whereas on the other hand, the human body may absorb and scatter the radiated energy intended to be directed into free space. The latter deteriorates the performance of the antenna. To alleviate these effects, researchers have adopted AMC structures in low-profile wearable antennas, which will act as a reflector for the radiated energy in specified frequencies. 

Through analyzing the metasurface antenna design have proposed in literature. The conventional method is that the near-zero reflection phase of the unit cell is set at around the resonance frequency by controlling the topologies of the AMC unit cells [20,61,64,65,83,84], which is also the main way to design a metasurface wearable antenn.

For the decrease of the SAR and improvement of the gain, another work in [63] proposed an AMC-backed printed Yagi–Uda antenna for on-body communication. The proposed antenna is made flexible using the combination of copper foil tape and polyester sheet. The antenna operates in the 2.4 GHz industrial, scientific, and medical (ISM) band. The printed Yagi-Uda is used to achieve a high gain, endfire radiation pattern, and decreased path loss. An EBG surface is employed to minimize the frequency detuning of the antenna and to reduce the specific absorption rate (SAR), while increasing the antenna gain when placed on the human body. The antenna gain increased by 60%, whereas SAR is decreased from 8.55 to 0.07 W/kg when the antenna is backed using the EBG. Despite being excellent in terms of gain and SAR, the size of the proposed antenna is still large and may not be suitable for some wearable applications. Alemaryeen et al. studied the effect of crumpling on textile coplanar waveguide (CPW) fed by a monopole antenna integrated with a flexible AMC structure [85]. Kwak et al. proposed a planar inverted-F antenna (PIFA) involved with an AMC structure for body SAR reduction, besides highlighting that an AMC structure without via can control the radiation pattern of the antenna and reduce the electromagnetic wave radiated towards the human body [86]. Agarwal et al. presented a technique of combining the endfire radiated wearable planar antenna with an AMC surface [87]. In this design, a single-layered AMC and a double-layered AMC are combined with a conventional planar Yagi antenna to change the direction of the radiation. Results showed that the double-layered AMC can improve the antenna performance in terms of front-to-back ratio, frequency detuning, radiation efficiency, and SAR. However, the bandwidth of 45 MHz of this antenna is not wide enough in practice. Metasurface structures are introduced in wearable antenna designs to provide a high degree of isolation from the human body and reduce the SAR values in the tissues. However, most periodic designs are electrically large and feature poor front to back ratio (FBR). An example is a low-profile wearable antenna integrated with a compact EBG structure proposed in [62], with a footprint of only 0.135 λ^2^. In addition, the significant size reduction compared to literature did not compromise its performance. It operates from 2.17 to 2.83 GHz with a bandwidth of 660 MHz (27%) and a gain of 7.8 dBi. More details are listed in Table 2.

**Figure 7:**
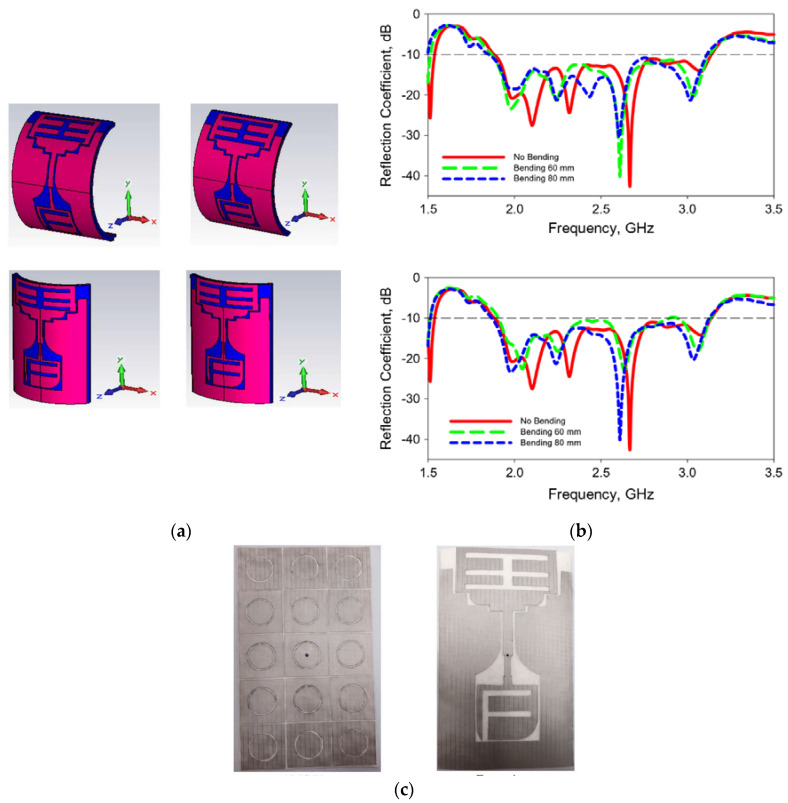
A wideband textile antenna with a ring-slotted AMC plane [90]. (**a**) The textile antenna bent at two different bending radii *r* and at two axes: *r* = 60 mm bent at *x*-axis, *r* = 80 mm bent at *x*-axis, *r* = 60 mm bent at *y*-axis, and *r* = 80 mm bent at *y*-axis. (**b**) Reflection coefficients of the antenna when bent at the *x*-axis and *y*-axis in (**a**). (**c**) The photo of the textile antenna: An artificial magnetic conductor (AMC) plane and a feeding structure.

For broadband, Lin et al. proposed a millimeter-wave band flexible fractal design with a self-similar window-like structure and an AMC as the reflector. This work also demonstrated for the first time that the performance of wearable antennas at mm-wave band, i.e., 20–40 GHz, can be improved using flexible AMC structures [88]. Besides that, Liu et al. studied a flexible windmill-shaped crossed-dipole antenna [89]. It featured a more compact structure with additional bandwidth over traditional dipoles, besides a more aesthetic outlook. Two narrowband resonances that are located close to each other have been combined to achieve a wider bandwidth in this metasurface-based antenna. In addition, a wideband wearable antenna, as shown in Figure 7c, which covers a bandwidth of 1.1 GHz (52%) was presented in [90], in which the four bending states are studied and the S11 curves are shown in Figure 7a,b. From the examples above, the AMC plane of this antenna plays a significant role in its wideband and backward suppression.

Apart from the common requirements of metasurfaces antenna, some special problems have addressed are reported in the literature. Chen et al. in 2016 proposed a lightweight, low-profile, and highly directive antenna for smartwatch applications [16]. It is reported that the properties of the AMC unit cell designed using the conventional method, in which the near-zero reflection phase of the unit cell is set at around the resonance frequency, are significantly different compared to the 2 × 2 array in practice. This is due to its properties being obtained from infinite-size structure simulations. To overcome this, the fractional factorial designs (FFDs) method is applied to analyze such a problem by considering the entire integrated structure. In addition, Kamardin et al. proposed a method to improve the transmission between on-body antennas using textile AMC waveguide sheets [91]. It was found that the AMC sheet actually acts as a subsidiary waveguide, which offers a new independent transmission path to minimize the transmission loss. Meanwhile, the enhancements of antenna structures via the application of metasurface have also be researched. Due to the bandgap characteristics of metasurfaces, such as in EBG structures, they can be effective in improving the isolation of a MIMO antenna system. Kim et al. used this characteristic for this purpose and demonstrated that the correlation coefficient can be reduced in a MIMO monopole antenna [92].

Besides textiles, inkjet printing has been applied to fabricate wearable antennas with metasurfaces [31,93]. These antennas are usually extremely lightweight and highly flexible while maintaining fabrication simplicity and low cost. However, such flexible structures are limited for practical wearable applications for humans, as they require stable performance in a severe environment—the additional flexibility results in performance deterioration and a decrease in mechanical robustness. 

To better complete complex, multi-functional metamaterial antennas, the general method hardly meets the need for efficient design. Thus, CMA theory is also introduced in the metasurface antenna study. Wen et al. proposed an improved alternative approach to designing AMC-based MIMO antennas in [74]. Instead of employing two antenna elements, a pair of degenerated characteristic modes (CMs) for one of the radiators is proposed in the design of an AMC-based MIMO antenna. A similar approach also is applied in [75,76], and it avoids the complicated design process of antennas and obtains the potential radiation performance of the complex metasurface antennas from the current mods.

**Figure 8:**
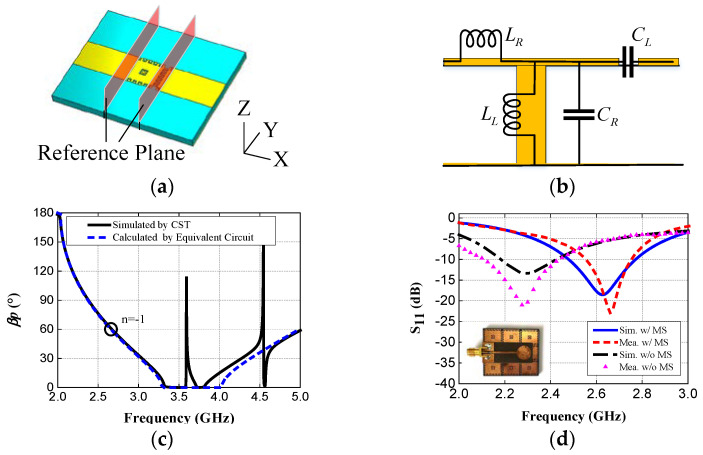
(**a**) Simulation model of dispersion of unit cell of the metasurface antenna. (**b**) The equivalent circuit of metasurface unit cell. (**c**) The dispersion curve simulated by CST and calculated by an equivalent circuit. (**d**) The photo of the compact, low-profile metasurface antenna and its S11 curve when feeding structure with/without metasurface plane [5].

**Figure 9:**
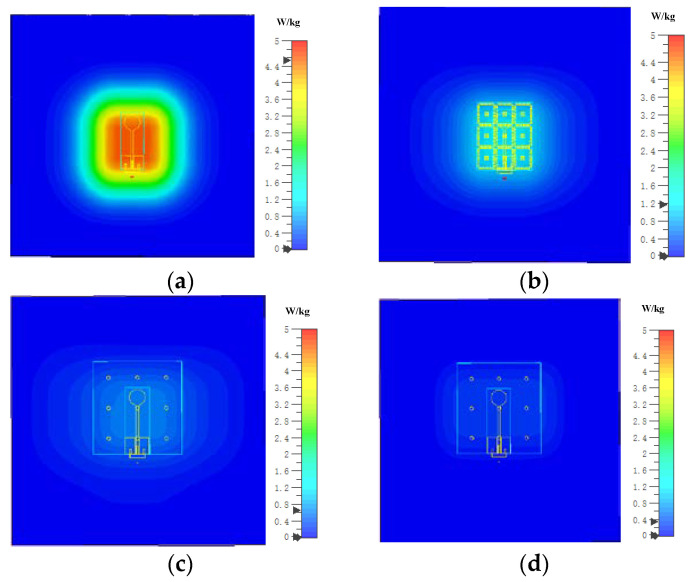
Simulated Specific Absorption Rate (SAR) of the metasurface antenna over the human body. (**a**) The feeding structure without metasurface at 2.65 GHz. (**b**) The feeding structure with metasurface at 2.65 GHz. (**c**) The dual band antenna at 2.5 GHz. (**d**) The dual band antenna at 3.65 GHz [5].

Researchers have found that finite metasurfaces not only functioned as reflecting ground planes, but also as the primary radiators or substrates with effective permittivities. Jiang et al. proposed a compact conformal wearable antenna that operates in the 2.36–2.4 GHz medical body area network band [94]. The antenna includes four I-shaped elements as an AMC and a UWB planar monopole antennas. This metasurface acts not only as a ground plane for improving the isolation between the radiator and the human body, but also as the main radiator. Compared with conventional metasurface-based antennas, this wearable antenna can significantly increase gain and decrease the coupling between the antenna and the human body. In fact, the monopole antenna only serves as a feed to excite the AMC structure, and its shape is not critical in impacting the antenna performance. According to this idea, the metasurface plane can be seen as a 2 dimension CRLH TLs, as shown in Figure 8, and the negative order resonance modes working in the left hand area are excited by the feeding structure, which reduces the size of the antenna drastically [5]. Figure 8c, d also show the simulated dispersion curve and S11 in different situations, and Figure 9 shows the simulated SAR of the metasurface antenna over the human body. However, the efficiency and the realized gain are also decreased due to its miniaturization. Meanwhile, in [95], a metasurface is considered as a substrate with a high relative permittivity when it is illuminated by a linearly-polarized wave.

### 3.2. Dual-Band Wearable Antennas with Metasurfaces

A number of researchers have been investigating dual-band wearable antennas based on metasurfaces [96,97,98,99,100,101,102,103,104]. This dual-band characteristic for wearable antennas with metasurface is usually realized by exciting the higher modes of the structures or by integrating different resonator shapes. The general design steps of such kind of wearable antennas are as follows [97]:

1. A dual-band antenna is first designed in the two frequency bands of interest.
2. An AMC is designed to operate within the same two bands as the antenna.
3. The AMC is integrated with the antenna and is optimized further in simulations via parametric study.
4. The optimized structure is fabricated and measured.

**Figure 10:**
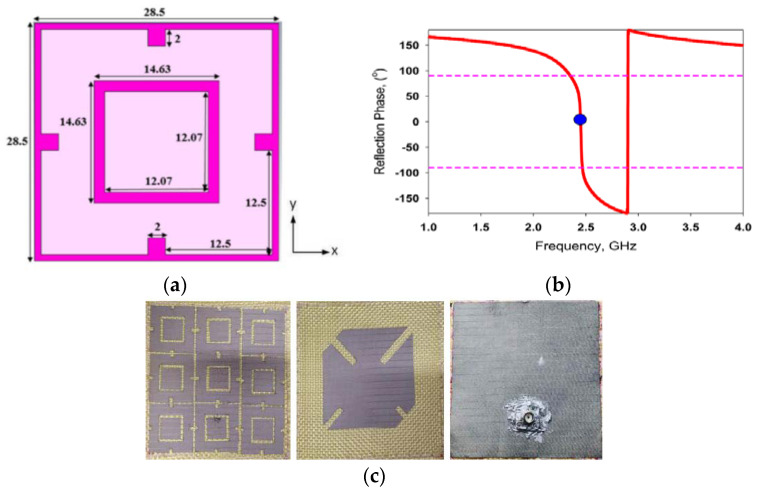
A dual-band wearable textile antenna [101]. (**a**) AMC unit cell dimensions of the AMC unit cell (in mm), (**b**) reflection phase of the AMC unit cell, and (**c**) fabricated prototype of the antenna.

**Figure 11:**
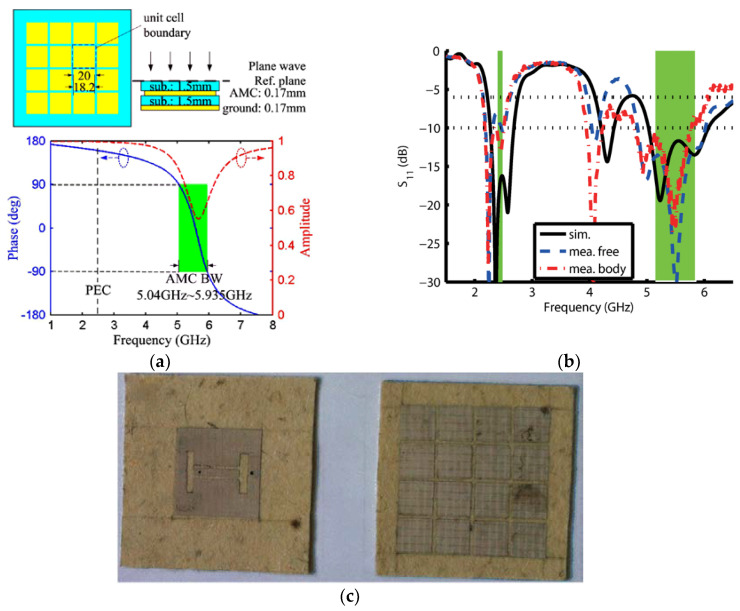
A low-profile dual-band textile antenna with artificial magnetic conductor plane [102]. (**a**) The AMC plane of the antenna and its reflection phase. (**b**) S11 curves of the dual-band antenna in free space and on the human body. (**c**) The photo of the wearable antenna.

Zhu et al. proposed a dual-band coplanar patch antenna integrated with an electromagnetic bandgap substrate [98,99]. The antenna structure is made using common clothing fabrics and operates in the 2.45 and 5 GHz wireless bands. The EBG consists of 3 × 3 elements and is shown to reduce radiation into the body by over 10 dB and improved gain by 3 dB. The performance of the antenna under different bending conditions and on-body conditions are also investigated. Based on this research, Bai further investigated the performance of a dual-band wearable antenna with an AMC under bending and crumpling conditions. Felt (with a relative permittivity of 1.39) is used as the substrate of the proposed antenna and Zelt conductive textile [100]. The performance of an integrated dual-band wearable antenna with AMC is studied under realistic crumpling conditions using shape deformations commonly found in clothing. Results showed that the influence of the crumpling on the antenna performances is acceptable for normal operation. A dual-band and dual-sense wearable antenna was designed, which operates in GPS and WLAN band with linear and circular polarization, and the AMC plane provides a decrease of backward and improvement of gain and bandwidth. The unite cell and its reflection phase are shown in Figure 10a,b, and the antenna prototype is shown in Figure 10c [101]. Next, Yan et al. proposed an efficiently radiating wearable antenna by replacing the normal metallic ground plane with an AMC plane, as shown in Figure 11. This AMC plane can be regarded as a PMC plane, whereas this antenna utilizes the patch as the radiator in the lower WBAN\WLAN band, and a slot dipole integrated on the patch to enable operation in the upper WLAN band [102], the simulation results are shown in Figure 11b. Such a principle avoids the general drawback commonly found in dual-band antennas with AMC plane such as bandwidth limitation and the increase in fabrication complexity. Similarly, Wang also presented a wearable dual-band antenna. The performance of the proposed antenna is studied via simulations and measurements when the antenna is located on different parts of a real human body and on different human bodies, including both man and woman [103]. Besides that, Velan et al. presented a dual-band wearable fractal-based monopole patch antenna integrated with an EBG structure [104]. The EBG structure reduces the radiation into the human body by more than 15 dB, besides reducing the effects of frequency detuning due to the human body. The performance of the antenna under bending, crumpling, and different on-body conditions have been studied. Finally, its specific absorption rate is also assessed to validate the antenna safety in wearable applications.

## 4. Reconfigurable Wearable Antennas with Metamaterials

**Figure 12:**
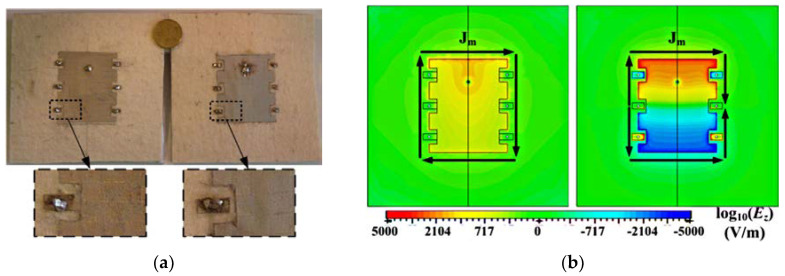
Radiation pattern-reconfigurable wearable antenna based on metamaterial structure [105]. (**a**) The photo of the wearable antenna. (**b**) Two electrical field distributions at 2.45 GHz in z-direction. The first distribution is Patch mode (*n* = +1) and second one is monopole mode (*n* = 0). The arrows in the figures represent the direction of the equivalent magnetic current.

**Figure 13:**
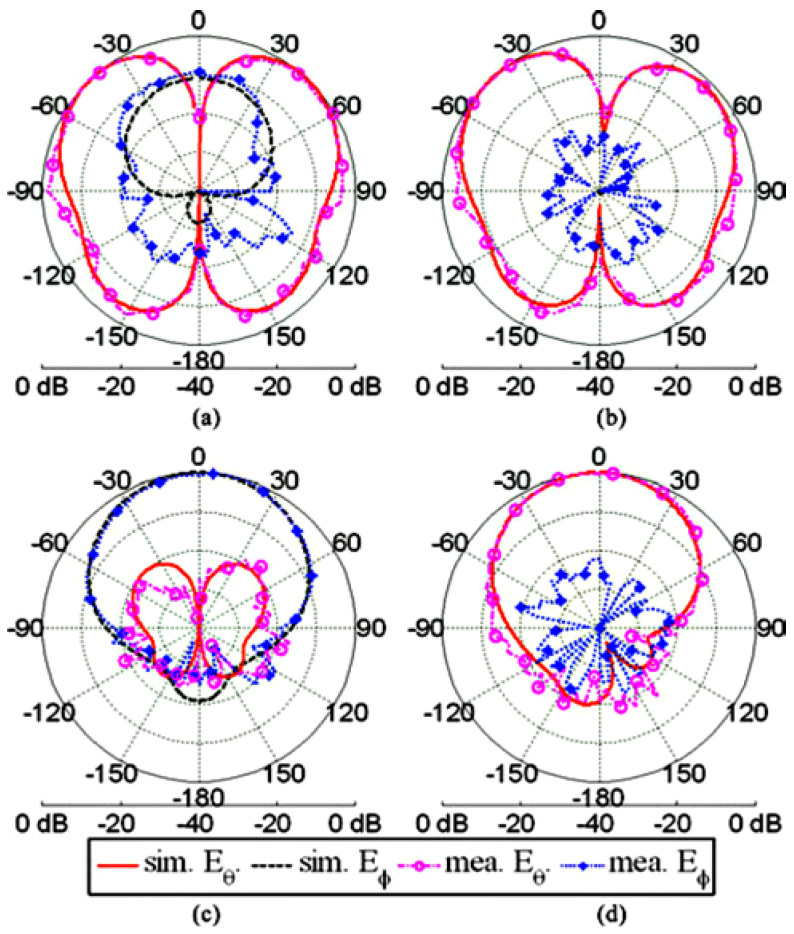
The radiation pattern of the antenna in [105]. Measured and simulated radiation patterns. (**a**) Monopole mode in xz-plane, (**b**) monopole mode in yz-plane, (**c**) patch mode in xz-plane, and (**d**) patch mode in yz-plane.

The space limitation in wearable antennas and the need for these antennas to operate in multiple wireless standards is spurring the development of reconfigurable antennas. Yan et al. presented a pattern-reconfigurable wearable antenna based on a metamaterial structure, as shown in Figure 12 [105]. This wearable antenna consists of three CRLH TL unit cells, which are capable of switching between the zero-order resonance and +1 resonance in the patch using switchable stubs connected using vias. The two states of this antenna operate in the same frequencies but radiate differently, providing a monopole-like or a patch like radiation pattern as shown in Figure 13, and the performances detail are listed in Table 3.

**Figure 14:**
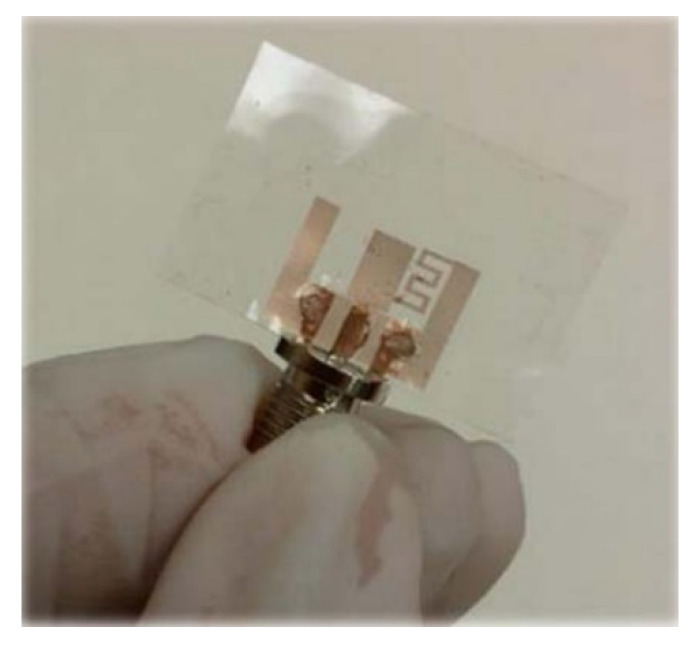
The photo of the mechanically reconfigurable electrically small antenna in [106]. The antenna made of flexible wire mesh and a frequency shift is generated in different stretch.

Besides that, Jang et al. proposed a method to fabricate a small semitransparent and stretchable antenna using a stretchable micromesh structure, as shown in Figure 14 [106]. This antenna consists of a 4.7 μm thick Cu mesh pattern and a PDMS layer as the substrate. The PDMS is flexible and optically transparent, and it can maintain the shape of a micromesh as well as protect the metal wire from mechanical damage when stretched. The increase in tensile strain reconfigures the resonant frequency of the antenna almost linearly from 2.46 to 2.94 GHz. However, this antenna suffers from low radiation efficiency due to the reduced surface currents flowing through the micromesh patch. 

Next, a wearable reconfigurable antenna with AMC structure consisting of a folded slot and a stub was proposed in [107]. This antenna operates between a single and dual-band mode, with two orthogonal polarizations controlled by the ON/OFF states of the PIN diodes. When the PIN diode is in the ON state, the stub is symmetrical with respect to the CPW feed line and does not radiate. This results in a single operating frequency of the antenna. When the PIN switch is in the OFF state, the asymmetrical stub with respect to the feed line causes the current on the stub to be redirected, producing dipole-like radiation. Both the slot and the stub resonate with orthogonal polarizations.

## 5. Conclusions

From this review, the design of wearable antennas is rather different from the design of conventional antennas. The main challenge is to ensure that the designed wearable antenna still operates with minimal coupling to the human body and under different deformations. Nonetheless, several studies have proven that the integration of metasurfaces onto the antenna design based can significantly improve their performance. This review also highlighted the recent progress in the literature on metamaterial-based wearable antennas, including the classification of the main approaches in their integration. As for the wearable antennas based on CRLH TLs, there are electrically small antennas based on ZOR consisting of ENR, MNR, and DNR modes and dual-band patch antennas, and their electromagnetic property, single-negative, or double-negative material parameter, may exhibit exciting performances, which can be utilized flexibly for different WBAN applications. For the wearable antennas based on metasurface, two methodologies have been presented: One approach is that the zero reflection phase of the unit cell of AMC is design at the resonance frequency so that the feeding antenna can be placed on the AMC reflection plane, decreasing the profile of the wearable antenna Another one is derived from the principle of CRLH TLs that the reflector is as a radiator by exciting the metasurface, and the main merit of it is decrease the size of the wearable antenna dramatically. Finally, three reconfigurable wearable antennas were described briefly. In summary, the radiation properties of these antennas can be improved by using metamaterials as follows:

- The radiation properties of wearable antennas can be enhanced by restraining the surface wave and the coupling between antennas and the human body.
- A low-profile of a wearable antenna can be realized by using the zero-reflection phase available from metasurfaces such as AMC structures.
- The bandwidth of wearable antennas can be broadened by loading reactive metasurfaces.
- The direction of radiation and level of gain can be controlled by modification of the field distributions and propagation directions.

Nonetheless, with the various requirements of today’s wireless communication systems, it should be emphasized that there is not a single type of wearable antenna that is capable of meeting all of the requirements simultaneously. However, metamaterial-based wearable antennas have been demonstrated to be capable of significantly improve the performance of antennas when applied to the human body compared with traditional antennas. It is foreseeable that wearable antennas would endeavor towards miniaturization, multifunction, multi-band frequency, and broadband in the future, and metamaterials-based antennas, which have unique properties, provide a new approach for these goals. Thus, a deeper understanding of the operation of metamaterials will result in more applications of such structures in future WBAN antennas.

## Figures and Tables

**Table 1:** Performances among the CRLH TL-based wearable antennas.

Ref.	Frequency (GHz)	Bandwidth	Gain (dBi)	Size (λ^2^)	SAR (W/kg)	Substrate (εr)
[53]	0.88/1.9	16%/29%	0.06/2.2	* 0.08(@0.88)	-	Substrate (2.41)
[57]	2.45/5.2	5.5%/11.2%	−3.5/6.6	0.16(@2.45)	0.012/0.25	Felt substrate (1.3)
[58]	2.45/5.4	3.8%/7.6%	−1.37/4.68	0.39(@2.45)		Felt substrate (1.3)
[77]	2.4	-	−5.2	0.01	-	RT/Duroid 5880 (2.2)
[78]	2.4	3%	-	0.04	-	Substrate (2.2)
[79]	2.4	6.5%	1.39	0.1	-	Rodgers RO 3003 (3.0)
[80]	2.4	2.5%	−7	-	-	RT/Duroid 5880 (2.2)

* (@0.88) means that the λ is the wavelength in free space at 0.88 GHz.

**Table 2:** Performances among the metasurface-based wearable antennas.

Ref.	Frequency (GHz)	Bandwidth	Gain (dBi)	Size (λ^2^)	SAR (W/kg)	Substrate (εr)
[5]	2.65	* 8.6% (@2.3) ** 11.5% (@2.65)	1.3 2.99	0.048 0.1	4.5 1.25	Substrate (2.65)
	2.45/3.65	13.6% (@2.2) 15.7%/2.3%	- 4.25/7.35	0.036 (@2.45) 0.2 (@2.45)	- 0.65/0.37	Substrate (2.65)
[61]	2.45	12% 18%	1.1 4.8	0.09 0.28	1.88 0.638	Kapton polyimide (3.5)
[62]	2.4	15% 27%	- 7.8	0.04 0.14	2.7 0.0138	Substrate (1.7)
[63]	2.4	14.5% 1.38%	3.41 8.53	1.22	8.85 0.69	Polyester (2.8)
[64]	2.45	- 4.88%	- 6.88	0.17	- 0.244	RT/duroid 5880 (2.2)
[65]	2.45	44.8% 25%	5.15 6.38	0.21 0.38	1.52 0.0072	Rogers 3850 (2.9)
[74]	2.45	- 3.6%	4.2	0.05	- 0.55	FR-4 (4.3)
[75]	5.5	- 15.8%	- 7.63	- 0.25	0.198	Substrate (2.7)
[76]	2.45	24% 16.3%	4.37 7.7	0.11 0.54	2.71 0.04	Substrate (1.6)
[83]	5.8	11% (@6.2) 10.7% (@5.8)	−3.83 −0.75	0.15 3.84	1.22 0.31	Leather substrate (2.3)
[84]	3.5/5.7/10/14	153% (2.1–16) 149% (2.3–16)	4.3 7.2	0.29 (@3.5)	- 0.1	FR-4 (4.3)
[85]	2.45	81% 16.3%	2.45 8.41	1.2 1.02	9.39 0.166	RO3003 (3.0)
[86]	1.97	2% 2%	2.8 4.6	0.006 0.05	1.4 0.7	Antenna (3.5) AMC (10.2)
[87]	2.45	0% 5%	4.17 2.32	0.16	4.2 0.714	Styrofoam (1)
[88]	20–40	61% (20–37.52) 64.4% (20.52–40)	7.4 10.3	2.5 (@28) 9.6 (@28)	- -	Substrate (2.2)
[89]	5.7–11	78% (5.25–12) 63% (5.7–11)	3.5 7.5	0.48 (@8) 1.28 (@8)	- -	Panasonic R-F770 (-)
[90]	2.5	42% 52%	0 3.38	1.38	- 0.025	Felt substrate (1.44)
[93]	2.45	8% 10%	−8.2 0.86	- 1.3	- -	Photo paper (-)
[94]	2.45	4.7% 5.5%	2.0 6.2	0.078 0.15	11.3 0.48	Rogers. RO3003 (3)
[95]	5.5	20% (5.5–6.75) 17%	5.2 6.7	0.38	- 0.43	Wool felt (1.2)
[96]	2.45/5.8	32%/25.8% 8%/27.5%	1.97/4.2 6.3/6.7	0.078 (@2.45) 0.25 (@2.45)	21.41/7.57 0.414/0.9	Felt substrate (1.22)
[97]	2.5/5.5	12%/41% 56%/32%	-	0.14 (@2.5) 0.50 (@2.5)	-	Felt substrate (1.22)
[99]	2.45/5.5	17%/16% 4%/12%	3.9/5.2 6.4/7.6	0.20 (@2.45) 0.96 (@2.45)	7.819/6.808 0.043/0.097	Felt substrate (1.38)
[101]	1.575/2.45	- 7.6%/5.5%	- 1.98/1.94	0.20 (@1.575)	- 0.78/0.71	Kevlar (1.66)
[102]	2.45/5.5	- 12%/16.3%	- 2.5/4	0.67 (@2.45)	- 0.019/0.009	Textile (1.3)
[103]	2.45/5.8	16.3%/3.4% 8%/6.8%	−4.1/2.33 5.2/7.7	0.05 (@2.45) 0.34 (@2.45)	8.99/4.08 0.7/0.71	Polyimide (3.5)
[104]	1.8/2.45	70% (1.3–2.7) 10.9%/5.08%	- 1–2	0.4 (@1.8) 0.81 (@1.8)	5.77/6.62 0.024/0.016	Jean (1.7)

* the antenna without metasurface; (@2.3) means that the λ is the wavelength in free space at 2.3 GHz; ** the antenna with metasurface.

**Table 3:** Performances among the reconfigurable wearable antennas with metamaterials.

Ref.	Frequenncy (GHz)	Bandwidth	Gain (dBi)	Size (λ^2^)	SAR (W/kg)	Substrate (εr)
[105]	2.4	0.086 (State1) 0.055 (State2)	2.9 4.5	0.64	0.05 0.01	Felt substrate (1.3)
[106]	2.92	10% (2.64–2.94)	−0.02	0.009	-	Tortuous Cu mesh/PDMS (2.8)
[107]	2.45/3.3	* - ** 7%/3%	2.6/0.6 6.2/3.0	***0.5 (@2.45)	2 0.29/-	RO3003 (3)

* the antenna without metasurface; ** the antenna with metasurface; *** (@2.45) means that the λ is the wavelength in free space at 2.45 GHz.
